# Histopathological Patterns of Cutaneous and Mucocutaneous Leishmaniasis Due to *L. aethiopica*

**DOI:** 10.1155/drp/5267606

**Published:** 2024-11-29

**Authors:** Abay Atnafu, Zewditu Chanyalew, Sofia Yimam, Meaza Zeleke, Shimelis Negussie, Selfu Girma, Aklilu Melaku, Menberework Chanyalew

**Affiliations:** ^1^Department of Communicable and Non Communicable Disease, Armauer Hansen Research Institute, Addis Ababa, Ethiopia; ^2^Department of Pathology, St. Paul's Hospital Millennium Medical College, Addis Ababa, Ethiopia; ^3^Dermatology Department, All African Leprosy, Tuberculosis, and Rehabilitation Training Center, Addis Ababa, Ethiopia

**Keywords:** cutaneous leishmaniasis, histopathology, mucocutaneous leishmaniasis, pattern

## Abstract

**Background:** Cutaneous leishmaniasis (CL) is an endemic disease in Ethiopia, mainly caused by *L. aethiopica*. Limited reports are available related to histopathological features of the skin lesion caused by *L. aethiopica*. This study aimed to analyze the histopathological features of CL due to *L. aethiopica*.

**Materials and Methods:** A similar cohort polymerase chain reaction (PCR) confirmed CL patients from a previous own study, who were prospectively enrolled from All Africa Leprosy, Tuberculosis and Rehabilitation Training (ALERT) Hospital Addis Ababa, Kela Health Center in Gurage Zone, Siliti Health Center in Silit zone of southern nations and nationalities, as well as Ankober Health Center in Amhara region was used for data analysis. The histopathology was analyzed by performing hematoxylin and eosin (H&E) staining to look for the presence of general and specific histopathology patterns of the disease. Descriptive statistics was utilized using SPSS version 26.0 (SPSS, Inc., Chicago, United States of America).

**Results:** Amastigotes were observed in skin biopsies of 29% (*n* = 2) mucocutaneous leishmaniasis (MCL) and 58% (*n* = 6) localized cutaneous leishmaniasis (LCL) patients. Diffused inflammatory cell infiltrate was observed in the dermal compartment of 77% (*n* = 20) samples while the remaining 23% (*n* = 6) had patchy or nodular inflammatory cell infiltrate. The dominant type of inflammatory cell infiltrate in the dermal compartments is macrophages and lymphocytes with a similar proportion, 23/26 (88.5%), followed by plasma cells, 21/26 (80.8%). Among all cases, 38.5% (*n* = 10) of them were categorized under the Type I pattern while Types IV and V patterns were reported in 26.9% (*n* = 7) and 34.6% (*n* = 9) of the remaining samples, respectively. The study found statistically significant correlations between necrosis and MCL (*p*=0.01), unorganized granulomas and LCL (*p*=0.04), and the presence of eosinophils and giant cell Langerhans with MCL (*p*=0.002 and *p* < 0.001, respectively).

**Conclusion:** In our study, the histopathological patterns of the CL caused by *L. aethiopica* were shown to have a dermal change that was characterized by a domination of diffused inflammatory cell infiltrate. Most of the cell types in the infiltrate were macrophages and lymphocytes. In addition, amastigote resided in the histiocyte with a varying degree of intensity, and both the organized and unorganized granulomas were shown with a considerable proportion.

## 1. Introduction

Cutaneous leishmaniasis (CL), an endemic disease in Ethiopia, is primarily caused by *Leishmania aethiopica* (L. *aethiopica*), though *Leishmania major* (L. *major*) and *Leishmania tropica* (L. *tropica*) have also been reported [1, 2]. Globally, an estimated 0.7–1 million people are affected annually, with 350 million at risk [3, 4]. Ethiopia is among the high-burden countries, reporting 20,000–30,000 cases per year [5].

The three species, *L. aethiopica*, *L. major*, and *L. tropica*, are the primary causative agents of CL in the Old World [6, 7]. Of these, *L. aethiopica* is predominantly found in Ethiopia and Eritrea, with a few reports from Kenya's border regions [4].

Diagnostic methods for CL include slit skin smear, culture, and polymerase chain reaction (PCR). While the culture method has lower sensitivity and PCR remains costly and less accessible, histopathology is a WHO-recommended diagnostic approach. Histopathologic diagnosis typically involves identifying amastigotes, along with characteristic changes in the skin's layers—the epidermis (outermost layer) and the dermis (deeper skin layer). In the dermis, characteristic infiltrates of macrophages, lymphocytes, plasma cells, and neutrophils, as well as granulomas, are commonly observed [8–11]. However, due to the limited geographic distribution of *L. aethiopica*, little is known about the histopathologic characteristics specific to CL caused by this species.

Although previous studies have analyzed histopathologic features of CL from various regions, data on the histopathology of Ethiopian CL caused by *L. aethiopica* are sparse. This lack of specific characterization makes it difficult to distinguish features attributable to *L. aethiopica* from those caused by other species [11, 12].

Therefore, this study aims to characterize the histopathologic features of CL caused by *L. aethiopica*, focusing on identifying Leishmania Donvani (LD) bodies, patterns of dermal inflammation (diffused or patchy), the presence of granulomas, and associated epidermal changes. In addition, our findings highlight panniculitis as a potential diagnostic marker in histopathologic examination of CL caused by *L. aethiopica*. In this study, the Leishmania species were confirmed as *L. aethiopica* using specific primers targeting *L. aethiopica* and a housekeeping gene.

## 2. Materials and Methods

### 2.1. Study Setting

A similar cohort of PCR confirmed CL patients [13], who were prospectively enrolled from All Africa Leprosy, Tuberculosis and Rehabilitation Training (ALERT) Hospital Addis Ababa, Kela Health Center in Gurage Zone, Siliti Health Center in Silit zone of southern nations and nationalities, as well as Ankober Health Center in Amhara region, was used for data analysis. Descriptive statistics and chi-squared test were performed using SPSS version 26 (SPSS, Inc., Chicago, United States of America). The study was conducted after obtaining approval from the ethical committee of the ALERT Center and Armauer Hansen Research Institute (AHRI), and the National Ethical Clearance Committee at the Federal Democratic Republic of Ethiopia Ministry of Science and Technology with ethical approval no. 310/227/2007, and it was approved on 30/05/2011. The ethical approval was renewed by the National Ethical Clearance Committee at the Federal Democratic Republic of Ethiopia Ministry of Science and Technology on 26/03/2016 with ref. number 3.10/003/2015. Written informed consent was obtained from study participants.

### 2.2. Sample Collection Procedures

Selecting the active edge of the lesion and making a nick, a slit skin smear was prepared. Using the blunt end of the scalpel blade, material scrapping from the wall of the nick was performed. The scabby component was removed from the lesion, and materials were scrapped from the base of the lesion for smear preparation. The prepared slides were left to air dry which were then fixed with methanol. The slides were then subjected to Giemsa staining. After being dried, the slides were examined under oil immersion using microscope for the presence of oval-shaped amastigote with the characteristic dot and dash appearance [14].

### 2.3. Biopsy Sample Collection

Biopsy sample was collected as previously described [13]. Using 3 mm disposable biopsy punch (Kai Europe GmbH, Kottendorfer Str. 5, 42697 Solingen, Germany), appropriate skin sample was collected form the margin of the lesion including the healthy and affected skin and bisected equally to be preserved half of it in 10% neutral-buffered formaldehyde (NBF) and the other half in 70% ethanol. The sample was sent to AHRI histopathology laboratory for further processing [14].

### 2.4. Slit Skin Smear (SSS)

Slit skin smear was collected following the standard protocol. The area around the lesion was cleaned with 70% alcohol and left air-dried. The edge of the lesion expected to contain amastigotes is scraped with a surgical blade to take appropriate sample excluding blood. A smear was prepared on a frosted-end slide and air-dried before proceeding to the staining procedure [15].

### 2.5. Giemsa Staining

The air-dried SSS was fixed with methanol to prevent it from being washed away during the staining procedure. The slides containing the smear were stained in a working Giemsa staining, prepared diluting the stock solution in 1:10 concentration, and filleted with filter paper. After the end of the staining time, the slides gently washed with running tap water, air-dried, and sent to microcopy room for further examination [15].

### 2.6. Tissue Processing, Embedding, and Sectioning

After 48 h of fixation in NBF, the tissue was processed overnight for a total of 11 h in a series of ethanol, xylene, and molten paraffin using Leica Biosystems automated tissue processor (Model: ASP-300S, SN: 00000006452). The next morning, the tissue was embedded using Leica Biosystems tissue embedding station (Model: HistoCore Arcadia H, SN:023). Tissue sections were prepared at 4 *μ*m thickness using Leica Biosystems Microtome (Model: RM2255, SN:11484) [16].

### 2.7. H&E Staining

The slides containing tissue sections were deparaffinized using a dry oven at 600°C for 30 min and in two changes of xylene for 10 min each, then cleared and rehydrated in a series of decreasing concentrations of alcohol and finally in tap water. The slide was stained for 8 min in Harris's hematoxylin reagent, destained in 0.5% acid–alcohol for 5 s and washed in running tap water. The counter staining was performed with 0.5% eosin for 1 min. After dehydration with alcohol and xylene, mounted with dibutylphthalate polystyrene xylene (DPX) mounting medium for histopathological examination [16].

### 2.8. Histopathological Examination

Different histopathological features were grouped as dermal reactions and epidermal changes. In dermal reactions, features other than the presence of LT bodies included the presence of plasma cell infiltrate with or without necrosis, granuloma formation, giant cells, lymphocytes, epithelioid cells, and polymorphonuclear leukocytes. The skin biopsies were evaluated by the dermatopathologist and histopathologist. Different types of inflammatory infiltrate including macrophage, lymphocyte, plasma cell, neutrophil, eosinophil, and giant cells were also reported and graded as absent, low, moderate, and high. The parasitic index was also categorized from 0 up to 6+ according to Ridley's parasitic index [14, 17].

The histopathological patterns were categorized as follows: Type I exudative-cellular reaction due to infiltration of histiocytes, lymphocytes, and plasma cells, without granuloma; Type II exudative-necrotic reaction, characterized by inflammatory infiltrate, and necrosis and no granulomatous response; Type III exudative and necrotic-granulomatous reaction (unorganized granuloma) corresponding to pattern described as chronic granulomatous inflammation with necrosis; Type IV exudative granulomatous reaction (unorganized granuloma) without necrosis characterized by the presence of an unorganized granulomatous reaction; Type V exudative tuberculoid reaction in which a typical tuberculoid granuloma (organized) is formed [18].

### 2.9. Culture

After appropriate cleaning of the active part of the lesion with 70% ethanol and air-dried for a moment, a surgical blade was ued to collect appropriate samples for culture with gentle scraping of the skin excluding blood. The sample was suspended in sterile locks media in the Nunc tube and sent to AHRI parasitology laboratory for culture. As soon as the sample arrived in the laboratory, it was cultured on Novy–MacNeal–Nicolle (NNN) and kept in an incubator at 24°C and followed for the next consecutive day to look for the growth of promastigotes [15].

### 2.10. DNA Extraction and PCR Amplification

The DNA extraction and PCR amplification protocol that was used for species confirmation was shown in our previous study [13]. DNA extraction from culture and biopsy samples was performed using the QIAamp DNA Mini Kit following the manufacturer's protocol. Briefly, Leishmania promastigotes were harvested and resuspended in Proteinase K ALT buffer, followed by vortexing and incubation. The sample was then mixed with ethanol, transferred to a QIAamp Spin Column, and washed with Buffer AW1 and AW2. Finally, the DNA was eluted using Buffer AE or distilled water and quantified using NanoDrop.

The HotStarTaq Plus Master Mix Kit from Qiagen and 100 ng of template DNA were used in this study. The master mix composition included Taq Polymerase (1 U) and 1 *μ*L of PCR buffer, Tris–HCl (10 mM), MgCl2 (pH 9.0, 1.5 mM), 50 mM KCl, a final concentration of 0.25 mM dNTPs, and 10 pmol each of forward and reverse primers. The volume was adjusted to 50 *μ*L using sterile distilled water.

Primer sets used in this study are as follows:

 
*L. aethiopica* species–specific primers
  V5F 5′-GGTGATGTGCCCGAGTGCA-3′
  V10R 5′-CGTGCACATCAGCACATGGG-3′
  Primers targeted for the housekeeping gene
 
*β*-actin F 5′-ATCTGGCACCACACCTTCTACAATGAGCTGCG-3′
 
*β*-actin R 5′-CGTCATACTCCTGCTTGCTGATCCACATCTGC-3′

The PCR process began with an initial denaturation step at 95°C for 5 min, followed by 30 cycles consisting of denaturation at 95°C for 1 min, annealing at 60°C for 2 min, and extension at 72°C for 2 min, followed by a final extension step at 72°C for 10 min. Ethidium bromide was incorporated into the PCR product for visualization, and the resulting samples were subjected to electrophoresis on a 1.5% agarose gel. Size determination was achieved using a one-kilobase plus ladder.

## 3. Result

### 3.1. Confirmation of *L. aethiopica* by Species-Specific Primers

**Figure 1:**
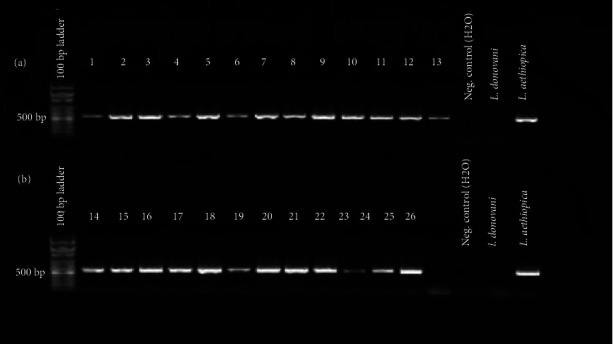
“PCR analysis of Leishmania isolates. DNA was extracted from Leishmania promastigote cultures that were grown on NNN medium. PCR by using *L. aethiopica*–specific primers, V5F and V10R was carried out. In (a) and (b), the first lanes represent 100 bp DNA marker. All lanes labeled with 1–26 are isolates from Ethiopian CL patients. The remaining lanes show H_2_0 control, *L. donovani* (reference number: MHOM/IN/80/DD8), and *L. aethiopica* reference strains (reference number: MHOM/ET/72/L100).”

The conventional PCR assay confirmed that all Leishmania isolates from the CL patients belong to *L. aethiopica*, as shown in Figure 1.

### 3.2. Histopathological Findings

The histopathology of 26 study participants was analyzed by H&E staining. Amastigote was observed in skin biopsies of 25% (*n* = 2) MCL and 61% (*n* = 11) LCL patients. One of the two parasite-positive samples from MCL patients has a 2+ load while the other one is 3+. Among the 11 samples from CL patients, four samples with 1+, three samples with 2+, two samples with 3+, one sample with 4+, and one sample with 6+ parasite index. The most common epidermal changes are hyperkeratosis/orthokeratosis accounting for 77% (*n* = 20) followed by follicular plugging 65% (*n* = 17). Ulceration was a less commonly reported histopathologic pattern within 8% (*n* = 2) of all cases followed by parakeratosis and atrophy (basal layer degeneration) comprising 19% (*n* = 5) and 23% (*n* = 6), respectively.  Table 1.

Diffused inflammatory cell infiltrate was observed in the dermis of 77% (*n* = 20) samples, while the remaining 23% (*n* = 6) had patchy or nodular inflammatory cell infiltrate. The dominant Type I of inflammatory cell infiltrate in the dermis are macrophages, 23/26 (88.5%), and lymphocyte, 23/26 (88.5%), followed by plasma cells, 21/26 (80.8%). Eosinophils are the rare cell population in the inflammatory infiltrate observed in a small number of skin biopsies. Among all cases, 38.5% (*n* = 10) of them were categorized under the Type I pattern while Types IV and V patterns were reported in 26.9% (*n* = 7) and 34.6% (*n* = 9) of the remaining samples, respectively, as shown in Table 1.

**Figure 2:**
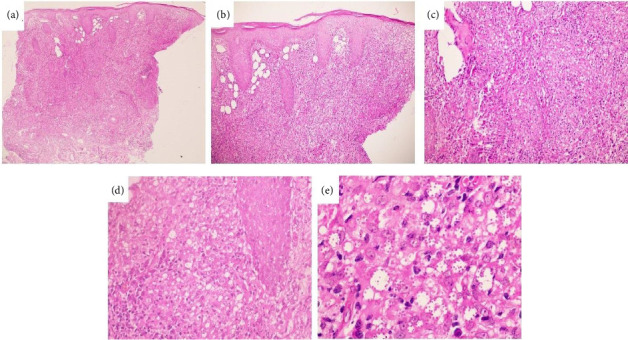
Histological features of skin biopsy from CL patients due to *L. aethiopica*. Diffuse dermal infiltrate ([a] 40x magnification). Dense dermal infiltrate of epithelioid histiocytes accompanied by lymphocytes and plasma cells. Epidermal atrophy in conjunction with infundibular hyperplasia and parakeratosis ([b] 100x magnification). Amastigotes and epidermal atrophy in conjunction with infundibular hyperplasia and parakeratosis. ([c, d] 200x and 400x magnification). Amastigotes readily evident in countless number ([e] 1000x magnification).

**Figure 3:**
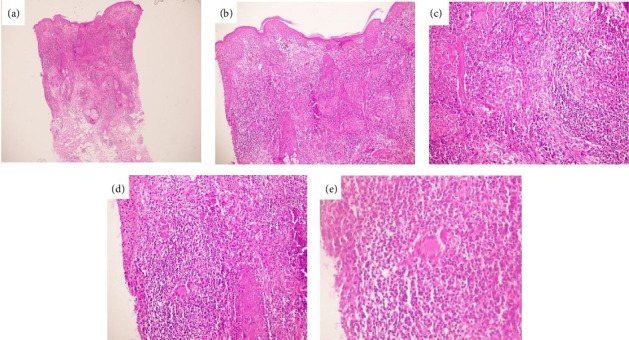
Confluent dermal nodules (a) 40x magnification. Epidermal atrophy in conjunction with infundibular hyperplasia and parakeratosis (b) 100x magnification. Dermal infiltrate composed of histiocytes, plasma cells, and lymphocytes accompanied by many multinucleated giant cells (c) 200x, (d) 400x, and (e) 1000x magnifications.

Dense diffused dermal infiltrate of epithelioid histiocytes accompanied by lymphocytes and plasma cells and amastigotes are shown in Figure 2. Confluent dermal nodules and dermal infiltrate composed of histiocytes, plasma cells, and lymphocytes accompanied by many multinucleated giant cells are shown in Figure 3.

In 61.5% (*n* = 16) of the skin sample, subcutaneous fat was missing showing that the proper sample collection procedure was compromised challenging the pathologists to analyze panniculitis in this type of sample. However, among the remaining properly collected samples (*n* = 10), the histopathology report prevailed panniculitis in 70% (*n* = 7) of the cases.

#### 3.2.1. Correlation of Clinical Form With the Parasite Index, the Type of Histopathological Pattern, and Dermal and Epidermal Changes

The presence of necrosis has shown a statistically significant correlation with MCL (*p* value = 0.01). On the other hand, the presence of unorganized granuloma was shown to be correlated with LCL (*p* value = 0.04). The presence of eosinophil and giant cell Langerhans appeared to be associated with MCL, and the correlation was statistically significant (*p* value = 0.002 and < 0.001, respectively). Among the epidermal changes, hyperkeratosis and follicular plugging are higher in number. Diffused inflammatory infiltrates are more commonly seen in LCL cases than patchy infiltrates with more macrophages, as shown in Supporting File 1.docx.

#### 3.2.2. Correlation of Duration and Number of the Lesion With Clinical Forms, Dermal and Epidermal Changes, and Type of Histopathological Patterns

In our study, the presence of patchy inflammatory cell infiltrate was shown to have a statistically significant correlation with a chronic lesion of more than 12 months duration (*p* value = < 0.0001). The presence of plasma cells was correlated with a single lesion, and the correlation was statistically significant (*p* value = 0.03). On the other hand, the presence of giant cell Langerhans was found to be correlated with the lesion of the duration of 3–12 months (*p* value = 0.04), as shown in Supporting File 2.docx

## 4. Discussion

CL in Ethiopia is mainly caused by *L. aethiopica* [19, 20]. In our setting, one of the commonly employed diagnostic tools is histopathology. However, there is a paucity of data narrating the brief characteristic of histopathological features especially when it comes to CL caused by *L. aethiopica*.

In a resource-limited country like Ethiopia, the alternative diagnostic tools such as the application of PCR technique are limited due to its expensive nature. One of the commonly employed diagnostic tools, histopathology, primarily relies on finding the suggestive features seen as dermal and epidermal alterations and in other instances on finding the amastigote stage of the parasite residing inside or outside histiocytes. In our study, we have observed a formation of granuloma in most of the CL cases. Similar report of granuloma formation has been shown by one study conducted in Sri Lanka [14]. In our study, majority of the cases have shown non–granulomatous reaction followed by organized and unorganized granulomatous reaction. A study conducted in Syria has reported a higher proportion of unorganized granuloma when compared with our study [21].

The dermis alteration involves the domination of diffused inflammatory infiltrate followed by patchy inflammatory cell infiltrate. Moderate to high infiltration of macrophage followed by lymphocyte, plasma cells, and neutrophils were also the characteristics of biopsies from CL patients in this study. However, giant cells, neutrophils, and eosinophils are less commonly observed. The formation of granuloma was reported to be an indication of an attempt of immune response to get rid of the invading pathogen [22].

Microscopical diagnosis of CL mainly relies on finding the amastigote stage of the parasite residing inside the histiocyte, and the amastigote may sometimes present outside the histiocyte [23, 24]. In our study, amastigotes were identified in 50% of the patients. This is similar to the previous studies, which is in the range of 38%–75% [25]. More than 90% of the samples have one or more epidermal changes in addition to inflammatory infiltrate although it is in the dermal compartment only. Therefore, the histology of skin biopsy is useful as an additional diagnostic method along with other parasite identification techniques. In this study, necrosis was only observed in 17 study participants. This result in line with the previous report has shown necrosis as an uncommon feature in Ethiopian CL patients [26].

The epidermal morphological alteration in CL is commonly reported in conjunction with dermal alterations [27]. In our study, atrophy, basal layer degeneration, and ulceration are the rare features among the study participants, which can also be used as differential diagnosis. A study conducted in Pakistan has shown a finding inconsistent with this study [28]. In our study, hyperkeratosis/orthokeratosis is a commonly observed feature in the study participants followed by follicular plugging, which can be used as differential diagnosis. A similar pattern of hyperkeratosis domination was reported by two studies. Follicular plugging was also reported to be a prominent finding from a study conducted in Turkey [14, 29]. An inconsistent finding from our study was reported by one study, indicating hyperkeratosis as a rare presentation of CL [30]. Inconsistency might be because the study was based on the new world region.

One of the rarely reported findings in histopathology of CL is panniculitis. The presence of panniculitis is a common feature in histopathology yet unrecognized despite its importance [31, 32]. Panniculitis refers to an inflammation of a subcutaneous tissue that may be generated due to several infectious conditions [33]. In this study, although in large proportion of skin sample (16/26) biopsy samples, subcutaneous is missing (most of the samples are not deep biopsies), 7/10 (70%) skin biopsies from CL patients have panniculitis. This finding is similar with the previous reports that have shown the presence of panniculitis among the study participants with CL [32, 34]. Therefore, this confirms that this characteristic could provide a clue and maybe considered as an additional histopathological feature to that of the dermal change of the CL caused by *L. aethiopica*.

In this study, participants of a small sample size were enrolled due to resource limitation. This hinders the opportunity to study further the presence of panniculitis and other histopathological feature at a larger scale.

## 5. Conclusion

In our study, the histopathological patterns of the CL caused by *L. aethiopica* were shown to have a dermal change that was characterized by a domination of diffused inflammatory cell infiltrate while the presence of the patchy inflammatory cell infiltrate was relatively low. Most of the cell types in the infiltration were macrophages and lymphocytes. Eosinophil was the rarely observed cell type in the inflammatory cell infiltrate. We also observed the presence of amastigote in half of the participants residing in the histiocytes. Similarly, we have also showed the presence of both the organized and unorganized granulomas where the organized granuloma predominates. In addition to the dermal changes, the spectrum of histopathological changes shown in our study was not limited to dermal changes rather accompanied by an epidermal change as well where a high domination of hyperkeratosis was showed. On another note, we observed the common yet unrecognized feature in histopathology of CL, panniculitis with considerable number. The presence of panniculitis could provide an additional clue and may be considered as an additional suggestive feature of CL along with that of dermal changes.

## Figures and Tables

**Table 1:** Semiquantitative analysis of histological finding in CL cases.

Epidermal findings	Numbers of cases (%)				
	Mild focal	Moderate	Severe	Total	Absent
Hyperkeratosis/orthokeratosis	2 (8%)	3 (12%)	16 (61.5%)	21(80.7%)	5 (19.2%)
Parakeratosis	3 (12%)	2 (7.7%)	1 (4%)	6 (23.1%)	20 (76.9%)
Ulcerated	0	3 (11.5%)	0	3 (11.5%)	88.5 (92%)
Acanthosis	3 (12%)	3 (12%)	9 (34.6%)	15 (57.7%)	11 (42.3%)
Pseudoepitheliomatous hyperplasia	0	4 (16%)	7 (28%)	11 (42%)	15 (58%)
Follicular plugging	0	1 (4%)	16 (62%)	17 (66%)	9 (35%)
Atrophy	0	1 (4%)	6 (23%)	7 (26.9%)	19 (73.1%)

**Number of cells in the inflammatory infiltrate**	**Numbers of cases (%)**				
	**Low**	**Moderate**	**High**	**Total present**	**Absent**

Macrophage	0	10 (38%)	13 (50%)	23 (88.5%)	3 (11.5%)
Lymphocyte	1 (4%)	11 (42%)	11 (42.3%)	23 (88.5%)	3 (11.5%)
Plasma cell	1 (4%)	8 (30.8%)	12 (46.2%)	21 (80.7%)	5 (19.2%)
Neutrophil	2 (8%)	3 (12%)	4 (15.4%)	9 (35%)	17 (65%)
Eosinophil	1 (4%)	2 (8%)	0	3 (12%)	23 (88%)
Giant cells	0	4 (15.4%)	0	4 (15.4%)	22 (84.6%)
Epitheloid cell	1 (4%)	10 (38%)	8 (31%)	19 (73%)	7 (27%)
Histopathological patterns	Type I	Type II	Type III	Type IV	Type V
Frequency (%)	10 (33.3)	0	0	7 (23.3)	9 (30)

## Data Availability

The underlying data are available upon request.

## Nomenclature

AHRI: Armauer Hansen Research Institute
CL: Cutaneous leishmaniasis
DNA: Deoxyribonucleic acid
dNTP: Deoxynucleotide triphosphate
DPX: Dibutylphthalate polystyrene xylene
H&E: Hematoxylin and Eosin
HCL: Hydrogen chloride
KCl: Potassium chloride
LCL: Localized cutaneous leishmaniasis
LT: Leishmania tropica
ALERT: All African Leprosy, Tuberculosis, and Rehabilitation Training Center
MCL: Mucocutaneous leishmaniasis
MgCl_2_: Magnesium chloride
NBF: Neutral-buffered formaldehyde
NNN: Novy–MacNeal–Nicolle
PCR: Polymerase chain reaction
SPSS: Statistical Package for Social Sciences
SSS: Slit-skin smear
USA: United States of America

## Supporting Information

Supporting Information 1 Supporting File 1: Correlation of clinical form with the parasite index, the type of histopathological pattern, and dermal and epidermal changes.

Supporting Information 2 Supporting File 2: Correlation of duration and number of the lesion with clinical forms, dermal and epidermal changes, and type of histopathological pattern.
