# Supplementary material for: Histopathological Patterns of Cutaneous and Mucocutaneous Leishmaniasis Due to L. aethiopica
Source: Dermatol Res Pract. 2024 Nov 29;2024:5267606. doi: 10.1155/drp/5267606 (PMC11623993; doi:10.1155/drp/5267606)
Supplement: Supporting Information 1 — Supporting File 1: Correlation of clinical form with the parasite index, the type of histopathological pattern, and dermal and epidermal changes. [file 5267606.f1.docx]

Supplementary file 1 Correlation of clinical form with the parasite index, the type of histopathological pattern, and dermal and epidermal changes

| Characteristics | | | Clinical form | | X^2^ | *P* value |
| --- | --- | --- | --- | --- | --- | --- |
|  |  |  | CL | MCL |  |  |
| Parasite index/load | | 1+ | 4 | 0 | 3.1 | *0.6* |
|  |  | 2+ | 3 | 1 |  |  |
|  |  | 3+ | 2 | 1 |  |  |
|  |  | 4+ | 1 | 0 |  |  |
|  |  | 6+ | 1 | 0 |  |  |
|  |  | Absent | 8 | 5 |  |  |
| Necrosis | | Present | 0 | 2 | 5.8 | *0.01** |
|  |  | Absent | 19 | 5 |  |  |
| Type of histopathological pattern | | Type 1 | 8 | 2 | 6.5 | *0.04** |
|  |  | Type 4 | 7 | 0 |  |  |
|  |  | Type 5 | 4 | 5 |  |  |
| Dermal changes | Diffused cell infiltration | Present | 13 | 7 | 2.8 | 0.09 |
|  |  | Absent | 6 | 0 |  |  |
|  | Cell infiltration patchy | Present | 2 | 0 | 0.8 | 0.4 |
|  |  | Absent | 17 | 7 |  |  |
|  | Macrophage | Present | 16 | 7 | 1.2 | 0.3 |
|  |  | Absent | 3 | 0 |  |  |
|  | Lymphocyte | Present | 16 | 7 | 1.2 | 0.3 |
|  |  | Absent | 3 | 0 |  |  |
|  | Plasma cell | Present | 15 | 6 | 0.2 | 0.7 |
|  |  | Absent | 4 | 1 |  |  |
|  | Neutrophil | Present | 5 | 4 | 2.1 | 0.1 |
|  |  | Absent | 14 | 3 |  |  |
|  | Eosinophil | Present | 0 | 3 | 9.2 | 0.002* |
|  |  | Absent | 19 | 4 |  |  |
|  | Epithelioid cell | Present | 12 | 7 | 3.5 | 0.06 |
|  |  | Absent | 7 | 0 |  |  |
|  | Giant cell Langerhans | Yes | 0 | 4 | 12.8 | <0.001* |
|  |  | No | 19 | 3 |  |  |
| Epidermal changes | Hyperkeratosis/Orthokeratosis | Present | 14 | 7 | 2.3 | 0.3 |
|  |  | Absent | 4 | 1 |  |  |
|  | Parakeratosis | Present | 5 | 1 | 0.2 | 0.6 |
|  |  | Absent | 14 | 6 |  |  |
|  | Ulcerated | Present | 2 | 1 | 0.07 | 0.8 |
|  |  | Absent | 17 | 6 |  |  |
|  | Acanthosis | Present | 9 | 6 | 3.9 | 0.13 |
|  |  | Absent | 10 | 1 |  |  |
|  | Pseudo epithelioimatous hyperplasia | Present | 7 | 4 | 0.7 | 0.4 |
|  |  | Absent | 11 | 3 |  |  |
|  | Follicular plugging | Present | 11 | 6 | 1.7 | 0.2 |
|  |  | Absent | 8 | 1 |  |  |
|  | Atrophy | Present | 5 | 2 | 0.5 | 0.8 |
|  |  | Absent | 14 | 5 |  |  |
