# Supplementary material for: Histopathological Patterns of Cutaneous and Mucocutaneous Leishmaniasis Due to L. aethiopica
Source: Dermatol Res Pract. 2024 Nov 29;2024:5267606. doi: 10.1155/drp/5267606 (PMC11623993; doi:10.1155/drp/5267606)
Supplement: Supporting Information 2 — Supporting File 2: Correlation of duration and number of the lesion with clinical forms, dermal and epidermal changes, and type of histopathological pattern. [file 5267606.f2.docx]

Supplementary file 2. Correlation of duration and number of the lesion with clinical forms, dermal and epidermal changes, and type of histopathological pattern

| Characteristics | | | Duration of lesion | | | X^2^ | *P* value | Number of lesions | | X^2^ | *P* value |
| --- | --- | --- | --- | --- | --- | --- | --- | --- | --- | --- | --- |
|  |  |  | Acute (<3 months) | Subacute (3-12 months) | Chronic (>12 months) |  |  | Single | Multiple |  |  |
| Clinical form | | CL | 0 | 17 | 1 | 2.9 | 0.2 | 13 | 6 | 1.4 | 0.2 |
|  |  | MCL | 1 | 5 | 2 |  |  | 3 | 4 |  |  |
| Dermal changes | Diffused cell infiltrate | Present | 1 | 18 | 1 | 3.8 | 0.1 | 12 | 8 | 0.09 | 0.8 |
|  |  | Absent | 0 | 4 | 2 |  |  | 4 | 2 |  |  |
|  | Patchy cell infiltrate | Present | 0 | 0 | 2 | 16.6 | <0.0001* | 1 | 1 | 0.01 | 0.7 |
|  |  | Absent | 1 | 22 | 1 |  |  | 15 | 9 |  |  |
|  | Macrophage | Present | 1 | 9 | 3 | 0.6 | 0.7 | 14 | 9 | 0.04 | 0.8 |
|  |  | Absent | 0 | 3 | 0 |  |  | 2 | 1 |  |  |
|  | Lymphocyte | Present | 1 | 19 | 3 | 0.6 | 0.7 | 14 | 9 | 0.04 | 0.8 |
|  |  | Absent | 0 | 3 | 3 |  |  | 2 | 1 |  |  |
|  | Plasma cell | Present | 1 | 18 | 2 | 0.6 | 0.7 | 15 | 6 | 4.5 | 0.03* |
|  |  | Absent | 0 | 4 | 1 |  |  | 1 | 4 |  |  |
|  | Neutrophil | Present | 1 | 8 | 0 | 3.5 | 0.2 | 6 | 3 | 0.1 | 0.7 |
|  |  | Absent | 0 | 14 | 3 |  |  | 10 | 7 |  |  |
|  | Eosinophil | Present | 0 | 3 | 0 | 0.6 | 0.7 | 2 | 1 | 0.04 | 0.8 |
|  |  | Absent | 1 | 19 | 3 |  |  | 14 | 9 |  |  |
|  | Epithelioid cell | Present | 1 | 15 | 3 | 1.7 | 0.4 | 11 | 8 | 0.4 | 0.5 |
|  |  | Absent | 0 | 7 | 0 |  |  | 5 | 2 |  |  |
|  | Giant cell Langerhans | Present | 0 | 19 | 3 | 6.1 | 0.04* | 2 | 2 | 0.3 | 0.9 |
|  |  | Absent | 1 | 3 | 0 |  |  | 14 | 8 |  |  |
| Type of histopathological patterns | | Type 1 | 0 | 7 | 3 | 7.1 | 0.1 | 7 | 3 | 1.7 | 0.4 |
|  |  | Type 4 | 0 | 7 | 0 |  |  | 5 | 2 |  |  |
|  |  | Type 5 | 1 | 8 | 0 |  |  | 4 | 5 |  |  |
| Epidermal changes | Hyperkeratosis | Present | 1 | 19 | 2 | 1 | 0.6 | 13 | 8 | 0.01 | 0.9 |
|  |  | Absent | 0 | 3 | 1 |  |  | 3 | 2 |  |  |
|  | Parakeratosis | Present | 0 | 6 | 0 | 1.4 | 0.5 | 2 | 3 | 1.2 | 0.3 |
|  |  | Absent | 1 | 16 | 3 |  |  | 14 | 7 |  |  |
|  | Ulcerated | Present | 1 | 2 | 0 | 8.2 | 0.02* | 2 | 1 | 0.04 | 0.8 |
|  |  | Absent | 0 | 20 | 3 |  |  | 14 | 9 |  |  |
|  | Acanthosis | Present | 1 | 11 | 3 | 3.5 | 0.2 | 7 | 7 | 1.7 | 0.2 |
|  |  | Absent | 0 | 11 | 0 |  |  | 9 | 3 |  |  |
|  | Pseudo epithelioimatous hyperplasia | Present | 1 | 7 | 3 | 6.4 | 0.04* | 5 | 6 | 2 | 0.1 |
|  |  | Absent | 0 | 15 | 0 |  |  | 11 | 4 |  |  |
|  | Follicular plugging | Present | 0 | 15 | 0 | 1.9 | 0.4 | 12 | 5 | 1.7 | 0.2 |
|  |  | Absent | 1 | 7 | 1 |  |  | 4 | 5 |  |  |
|  | Atrophy | Present | 0 | 6 | 0 | 1.4 | 0.5 | 4 | 2 | 0.09 | 0.8 |
|  |  | Absent | 1 | 16 | 3 |  |  | 12 | 8 |  |  |
